# Hydroxychloroquine prescription trends and predictors for excess dosing per recent ophthalmology guidelines

**DOI:** 10.1186/s13075-018-1634-8

**Published:** 2018-07-05

**Authors:** April M. Jorge, Ronald B. Melles, Yuqing Zhang, Na Lu, Sharan K. Rai, Lucy H. Young, Karen H. Costenbader, Rosalind Ramsey-Goldman, S. Sam Lim, John M. Esdaile, Ann E. Clarke, M. B. Urowitz, Anca Askanase, Cynthia Aranow, Michelle Petri, Hyon Choi

**Affiliations:** 1 Division of Rheumatology, Allergy, and Immunology, Department of Medicine, Massachusetts General Hospital, Harvard Medical School, 55 Fruit Street, Bulfinch 165, Boston, MA 02114 USA; 2 Department of Ophthalmology, Kaiser Permanente, Redwood City, CA USA; 3 Department of Ophthalmology, Massachusetts Eye and Ear Infirmary, Harvard Medical School, Boston, MA USA; 4 Division of Rheumatology, Immunology, and Allergy, Brigham and Women’s Hospital, Harvard Medical School, Boston, MA USA; 5 Rheumatology Division, Feinberg School of Medicine, Northwestern University, Chicago, IL USA; 6 Division of Rheumatology, Emory University School of Medicine, Atlanta, GA USA; 7 Arthritis Research Canada, Richmond, BC Canada; 8 Division of Rheumatology, University of Calgary, Calgary, AB Canada; 9 Centre for Prognosis Studies in the Rheumatic Diseases, University of Toronto, Toronto Western Hospital, Toronto, ON Canada; 10 Rheumatology, Columbia University College of Physicians & Surgeons, New York, NY USA; 11 Autoimmune and Musculoskeletal Disease, The Feinstein Institute for Medical Research, Manhasset, NY USA; 12 Division of Rheumatology, Johns Hopkins University School of Medicine, Baltimore, MD USA

**Keywords:** DMARDs, Epidemiology, Quality of care, Rheumatoid arthritis, Systemic lupus erythematosus

## Abstract

**Background:**

Hydroxychloroquine (HCQ) retinopathy may be more common than previously recognized; recent ophthalmology guidelines have revised recommendations from ideal body weight (IBW)-based dosing to actual body weight (ABW)-based dosing. However, contemporary HCQ prescribing trends in the UK remain unknown.

**Methods:**

We examined a UK general population database to investigate HCQ dosing between 2007 and 2016. We studied trends of excess HCQ dosing per ophthalmology guidelines (defined by exceeding 6.5 mg/kg of IBW and 5.0 mg/kg of ABW) and determined their independent predictors using multivariable logistic regression analyses.

**Results:**

Among 20,933 new HCQ users (78% female), the proportions of initial HCQ excess dosing declined from 40% to 36% using IBW and 38% to 30% using ABW, between 2007 and 2016. Among these, 47% of women were excess-dosed (multivariable OR 12.52; 95% CI 10.99–14.26) using IBW and 38% (multivariable OR 1.98; 95% CI,1.81–2.15) using ABW. Applying IBW, 37% of normal and 44% of obese patients were excess-dosed; however, applying ABW, 53% of normal and 10% of obese patients were excess-dosed (multivariable ORs = 1.61 and 0.1 (reference = normal); both *p* < 0.01). Long-term HCQ users showed similar excess dosing.

**Conclusion:**

A substantial proportion of HCQ users in the UK, particularly women, may have excess HCQ dosing per the previous or recent weight-based guidelines despite a modest decline in recent years. Over half of normal-BMI individuals were excess-dosed per the latest guidelines. This implies the potential need to reduce dosing for many patients but also calls for further research to establish unifying evidence-based safe and effective dosing strategies.

**Electronic supplementary material:**

The online version of this article (10.1186/s13075-018-1634-8) contains supplementary material, which is available to authorized users.

## Background

Hydroxychloroquine (HCQ) is commonly prescribed in patients with systemic lupus erythematosus (SLE) and remains a cornerstone of lupus care today [1, 2], and it is also often used in the management of rheumatoid arthritis (RA) and other rheumatic conditions. Since the landmark trial that showed HCQ discontinuation led to a nearly threefold higher risk of lupus exacerbation [3], many subsequent studies have reported wide-ranging benefits of HCQ, including improved survival, reduced disease activity and damage accumulation, and a lower risk of pregnancy complications, venous thromboembolism, dyslipidemia, and insulin resistance among patients with SLE [4–7]. In RA, the efficacy of triple therapy (which includes HCQ) is proven to be similar to that of etanercept [8], while being much more cost-effective [9]. This profile will likely remain attractive worldwide, even in the era of modern biologic agents.

Although HCQ is generally well-tolerated, the major long-term adverse event is vision-threatening toxic retinopathy [10, 11]. Rates of HCQ retinopathy were historically considered low (< 2%) [12–15]. Previously, the 2009 Royal College of Ophthalmologists (RCO) and 2011 American Academy of Ophthalmology (AAO) guidelines each recommended a maximum safe dose of 6.5 mg/kg/day of ideal body weight (IBW) (up to a maximum of 400 mg daily) to minimize the risk of retinopathy [2, 16, 17]. However, in 2016, the AAO updated their maximum daily dose recommendation to 5 mg/kg/day actual body weight (ABW), based largely on a retrospective study based in Kaiser Permanente Northern California (KPNC) [18–20]. This study utilized modern, sensitive screening methods that can identify early stages of retinopathy and identified an overall prevalence of HCQ retinopathy of 7.5%, over three times higher than previous estimates [14, 21, 22]. The rate of retinopathy was considerably higher with HCQ doses over 5.0 mg/kg ABW [18]. Following the KPNC study, there has been renewed concern in the USA about the appropriate dosing of HCQ, reflected in the latest AAO guidelines [15, 19, 23, 24]. Despite the previous IBW-based recommendation [16], there may have been less concern about HCQ retinopathy in the UK than in the USA, as the RCO had not recommended routine HCQ retinopathy screening prior to 2017 [16, 25–27]. To that end, to what extent recent HCQ prescriptions would exceed dosing recommended by ophthalmology guidelines remain unknown.

To understand recent prescribing trends in the UK and to elucidate the predictors of potential excess dosing, we assessed HCQ prescribing patterns in relation to the previous and latest HCQ dosing guidelines [16, 17, 20, 26] over a recent 10-year period in a UK general population database.

## Methods

### Data source

Our study cohort was derived from The Health Improvement Network (THIN), an electronic medical record (EMR) database which represents 6.2% of the UK population, including over 11 million patients. THIN database is representative of the general UK population in terms of demographics, lifestyle factors, and healthcare utilization [28]. Healthcare information includes general practitioner visits, specialist referrals, diagnoses from hospital admissions and specialists, medications, and laboratory results. The specific diagnoses are recorded by the Read code classification system, which is the standard nomenclature of clinical terms used by the National Health Service in describing clinical diagnoses [29]. Medication prescriptions are recorded by the Multifunctional Standardized Lexicon for European Community Language (MULTILEX) classification system [30].

### Study population and design

We identified all subjects age 18 years or older within THIN with incident HCQ prescriptions between 1 January 2007 and 31 December 2016. We divided these cohorts into five 2-year blocks each, based on index date, determined by the date of first documented HCQ prescription. We required ≥1 year of subject inclusion in THIN prior to the index date to be considered an incident prescription.

### Assessment of covariates

We determined information from the most recent available data prior to the index date on age, sex, lifestyle (i.e., smoking), and anthropometric characteristics (i.e., height and body weight). We calculated body mass index (BMI) and IBW using the commonly used Devine formula (for women, 45.5 kg + 2.3 × height in inches over 60 in. and for men, 50 kg + 2.3 × height in inches over 60 in.) [30, 31], as was also used in the recent KPNC paper [18]. We identified the indication for HCQ use by Read diagnosis codes. We also determined baseline comorbidities (i.e., diabetes mellitus and chronic kidney disease (CKD) stage ≥ 3) and medication use (i.e., tamoxifen, which has been previously implicated in increased risk of HCQ retinopathy) [18].

### Assessment of outcomes

We identified the incident HCQ prescription dose for the primary analysis. We also obtained overall (incident and renewal prescriptions) HCQ prescription dose, assessing the first prescription dose in a given calendar year for each subject. We classified the weight-based excess HCQ dose according to recommended safe doses per IBW (i.e., > 6.5 mg/kg) [16, 27], and per ABW (i.e., > 5.0 mg/kg) [18, 19]. We also examined HCQ daily dose categories (i.e., 100 mg, 200 mg, 300 mg, 400 mg, 500 mg, and 600 mg), rounding to the nearest 100 mg (or one half tablet) per day, given that this medication is only available in 200 mg tablets.

To examine the dosing trends according to the duration of use, we obtained the initial HCQ prescription dose and the HCQ prescription dose at 5 years of treatment for a subgroup of subjects with at least 5 years of HCQ use. We similarly classified their 1st and 5th year prescription doses according to recommended doses per ABW and IBW.

### Statistical analysis

We compared the baseline characteristics of individuals at the index date of their incident HCQ prescriptions according to calendar year categories. We calculated the proportion of incident HCQ prescriptions exceeding either IBW or ABW maximum safe dose over the five 2-year cohort blocks and described the secular trends. We examined the relationship between age, sex, BMI, CKD, and indication for HCQ use and the risk of prescribed HCQ dose exceeding each safe dose for IBW or ABW, respectively, using a multivariable logistic regression model. The final multivariable model was adjusted for age, sex, BMI, CKD, indication for HCQ use, smoking, tamoxifen use, diabetes mellitus, and calendar year.

We calculated median and interquartile ranges for initial HCQ dose per IBW and ABW at 6-month intervals between 2007 and 2016 and performed quantile regression to assess the median value trends. Furthermore, we calculated the proportion of different HCQ dose categories (i.e., 600 mg, 500 mg, 400 mg, 300 mg, 200 mg, and 100 mg) comprising overall initial HCQ doses over this same timeframe. We also calculated the proportion of these dose categories comprising overall (incident and renewal) HCQ doses over this timeframe.

## Results

During the 10-year period between 2007 and 2016, 20,933 individuals initiated HCQ (Table 1). The majority were female (78%). The mean age was 55.6 years and mean BMI was 27.7 kg/m^2^. RA was the most common indication for HCQ use (56%), 2257 subjects (11%) had SLE, and 1572 (8%) had CKD ≥ stage 3.

**Table 1:** Baseline characteristics of hydroxychloroquine incident users by time period

	Time period of initial prescription					
Characteristics	2007–2008	2009–2010	2011–2012	2013–2014	2015–2016	All
	(*n* = 3297)	(*n* = 4151)	(*n* = 4927)	(*n* = 4944)	(*n* = 3614)	(*n* = 20,933)
Sex (% female)	2617 (79)	3267 (79)	3822 (79)	3846 (78)	2751 (76)	16,303 (78)
Mean age, years (SD)	53.7 (15)	54.9 (15)	55.7 (15)	56.2 (15)	56.9 (15)	55.6 (15)
BMI, kg/m^2^ (mean +/− SD)	27.3 (6)	27.4 (6)	27.6 (6)	27.8 (6)	28.4 (6)	27.7 (6)
BMI category (%)						
Underweight (BMI < 18.5)	81 (3)	89 (2)	95 (2)	90 (2)	68 (2)	424 (2)
Normal (BMI 18.5 – < 25)	1096 (37)	1371 (36)	1623 (36)	1553 (34)	1054 (31)	6697 (35)
Overweight (BMI 25 – < 30)	942 (32)	1274 (34)	1530 (34)	1527 (34)	1139 (33)	6412 (33)
Obese (BMI 30+)	815 (28)	1053 (28)	1271 (28)	1390 (31)	1157 (34)	5686 (30)
CKD (≥ stage 3)	213 (7)	344 (8)	366 (7)	366 (7)	283 (8)	1572 (8)
Tamoxifen use	26 (1)	47 (1)	66 (1)	69 (1)	54 (2)	262 (1)
Diabetes mellitus (%)	228 (7)	370 (9)	483 (10)	552 (11)	517 (14)	2150 (10)
Smoking status (% current smoker)	696 (22)	881 (22)	977 (20)	953 (20)	645 (18)	4152 (20)
Indication for HCQ (%)						
RA/inflammatory arthritis	1745 (53)	2352 (57)	2794 (57)	2863 (58)	2001 (56)	11,749 (56)
SLE	459 (14)	462 (11)	481 (10)	415 (8)	283 (8)	2100 (10)
Systemic autoimmune rheumatic disease^a^	117 (4)	156 (4)	183 (4)	174 (4)	148 (4)	778 (4)
Primary dermatologic disease	282 (9)	384 (9)	489 (10)	5142 (11)	486 (13)	2183 (10)
All other indications	700 (21)	797 (19)	980 (20)	950 (19)	696 (19)	4123 (20)

*BMI* body mass index, *CKD* chronic kidney disease, *HCQ* hydroxychloroquine, *SLE* systemic lupus erythematosus, *RA* rheumatoid arthritis

^a^Systemic autoimmune rheumatic disease category excludes SLE and RA

**Fig. 1:**
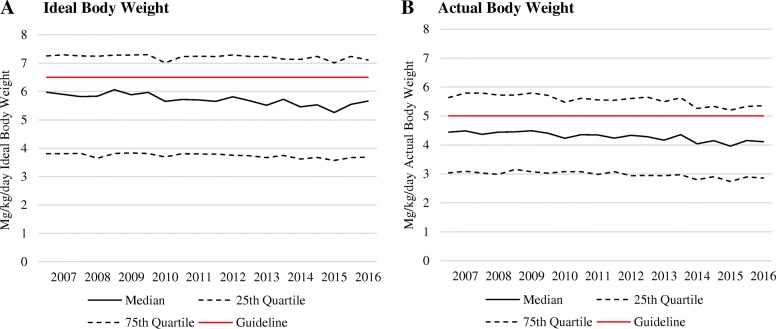
Trends of incident hydroxychloroquine prescription dose per ideal body weight and actual body weight (2007–2016). Median and inter-quartile ranges of incident hydroxychloroquine prescription dose, calculated per mg/kg of ideal body weight and per mg/kg of actual body weight, in relation to published guideline-recommendations

**Fig. 2:**
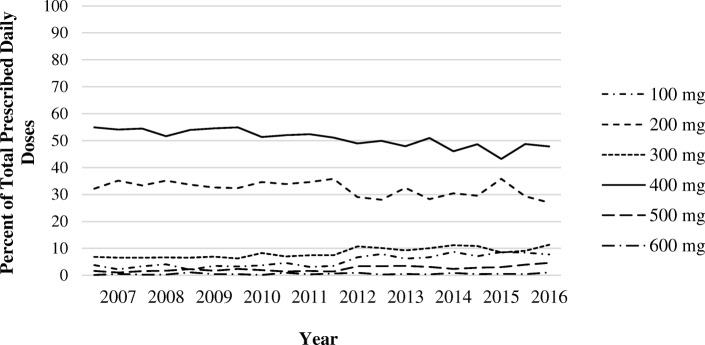
Incident hydroxychloroquine prescription dose trends. Proportion of incident hydroxychloroquine prescriptions in each dosing category over time, between 2007 and 2016

Between 2007 and 2016, proportions of initial HCQ excess dosing declined from 40% to 36% per IBW-based dosing (multivariable OR between 2007 and 2008 and between 2015 and 2016, 0.85 (95% CI 0.76–0.95)) and from 38% to 30% per ABW-based dosing (multivariable OR, 0.76 (95% CI 0.68–0.86)) (Table 2). Correspondingly, the median HCQ dose per IBW declined from 6.0 mg/kg to 5.7 mg/kg during the study period, and the median HCQ dose per ABW declined from 4.4 mg/kg to 4.1 mg/kg (both *p* values < 0.01) (Fig. 1). Furthermore, the proportions of initial prescribed daily dose categories of HCQ changed over time, with a slightly lower proportion of 400 mg per day prescribed in recent years and an increase in the relative proportions of 100 mg, 300 mg, and 500 mg doses, as shown in Fig. 2. These trends remain similar in our analyses that include subsequent doses after initial doses (Additional file 1: Figure S1). Furthermore, when we limited our analyses to those with body weight measured within 1 year prior to index date, our result did not change materially.

**Table 2:** Initial hydroxychloroquine prescription dose in relation to dosing recommendations

Characteristics	Dose > 6.5 mg/kg/day, ideal body weight (prior recommendation)			Dose > 5 mg/kg/day, actual body weight (latest recommendation)		
	Number (%)	Crude OR (95% CI)	Multivariable^a^ OR (95% CI)	Number (%)	Crude OR (95% CI)	Multivariable^a^ OR (95% CI)
Year of initial prescript						
2007–2008	1099 (40)	1.00 (Reference)	1.00 (Reference)	1055 (38)	1.00 (Reference)	1.00 (Reference)
2009–2010	1406 (39)	0.98 (0.88–1.08)	1.00 (0.89–1.11)	1348 (38)	0.97 (0.88–1.08)	0.99 (0.89–1.11)
2011–2012	1597 (38)	0.92 0.83–1.02)	0.95 (0.85–1.05)	1519 (36)	0.91 (0.82–1.00)	0.92 0.83–1.03)
2013–2014	1511 (37)	0.88 (0.80–0.97)	0.90 (0.76–1.00)	1408 (35)	0.84 (0.76–0.93)	0.88 (0.79–0.99)
2015–2016	1073 (36)	0.84 (0.76–0.94)	0.85 (0.76–0.95)	901 (30)	0.70 (0.62–0.78)	0.76 (0.68–0.86)
Sex						
Male	268 (7)	1.00 (Reference)	1.00 (Reference)	998 (25)	1.00 (Reference)	1.00 (Reference)
Female	6418 (47)	11.71 (10.30–13.32)	12.52 (10.99–14.26)	5233 (38)	1.76 (1.62–1.90)	1.98 (1.81–2.15)
Age						
≤ 55 years	3647 (38)	1.00 (Reference)	1.00 (Reference)	3453 (36)	1.00 (Reference)	1.00 (Reference)
> 55 years	3039 (38)	1.00 (0.94–1.08)	1.23 (1.15–1.32)	2778 (35)	0.95 (0.89–1.01)	1.18 (1.10–1.27)
BMI (kg/m^2^)						
Underweight (< 18.5)	104 (28)	0.74 (0.59–0.93)	0.66 (0.52–0.84)	176 (48)	0.82 (0.66–1.01)	0.78 (0.63–0.97)
Normal (18.5 – < 25)	2114 (35)	1.00 (Reference)	1.00 (Reference)	3215 (53)	1.00 (Reference)	1.00 (Reference)
Overweight (25 – < 30)	2134 (36)	1.07 (1.00–1.12)	1.28 (1.19–1.39)	2319 (39)	0.58 (0.54–0.63)	0.61 (0.57–0.66)
Obese (≥ 30)	2334 (44)	1.51 (1.40–1.63)	1.61 (1.49–1.75)	521 (10)	0.10 (0.09–0.11)	0.10 (0.09–0.11)
Smoking						
Current smoker	1245 (36)	0.89 (0.83–0.96)	1.06 (0.98–1.16)	1294 (37)	1.10 (1.02–1.19)	1.03 (0.95–1.13)
Non-smoker	5426 (39)	1.00 (Reference)	1.00 (Reference)	4924 (35)	1.00 (Reference)	1.00 (Reference)
CKD						
CKD stage > 3	516 (37)	0.97 (0.86–1.08)	0.88 (0.73–1.00)	429 (31)	0.81 (0.72–0.91)	0.87 (0.76–0.99)
No CKD	6170 (38.1)	1.00 (Reference)	1.00 (Reference)	5802 (36)	1.00 (Reference)	1.00 (Reference)
Diabetes						
Yes	726 (38)	0.99 (0.90–1.09)	1.05 (0.94–1.17)	483 (25)	0.58 (0.52–0.65)	0.92 (0.81–1.03)
No	5960 (38)	1.00 (Reference)	1.00 (Reference)	5748 (37)	1.00 (Reference)	1.00 (Reference)
Tamoxifen use						
Yes	113 (51)	1.67 (1.29–2.18)	1.16 (0.89–1.52)	94 (42)	1.32 (1.01–1.73)	1.16 (0.87–1.55)
No	6573 (38)	1.00 (Reference)	1.00 (Reference)	6137 (35)	1.00 (Reference)	1.00 (Reference)
Indication for HCQ						
RA/inflammatory arthritis	3651 (36)	1.00 (Reference)	1.00 (Reference)	3491 (35)	1.00 (Reference)	1.00 (Reference)
SLE	628 (37)	1.03 (0.93–1.15)	0.84 (0.75–0.94)	590 (35)	1.04 (0.93–1.17)	0.87 (0.77–0.98)
SARD	287 (43)	1.34 (1.14–1.56)	1.07 (0.91–1.26)	272 (41)	1.29 (1.10–1.51)	1.09 (0.92–1.30)
Primary dermatologic disease	745 (41)	1.23 (1.11–1.36**)**	1.11 (0.99–1.25)	655 (36)	1.04 (0.95–1.15)	1.12 (0.99–1.26**)**
Other	1375 (41)	1.24 (1.14–1.34)	1.16 (1.06–1.27)	1223 (37)	1.10 (1.01–1.19)	1.14 (1.04–1.25)

BMI body mass index, CKD chronic kidney disease, SLE systemic lupus erythematosus, RA rheumatoid arthritis, SARD systemic autoimmune rheumatic disease

^a^Multivariable odds ratios are adjusted for age, sex, BMI, CKD, indication for hydroxychloroquine use, smoking, tamoxifen use, diabetes mellitus, and calendar year

Using IBW-based recommended dose, 47% of women and 7% of men had excess dosing, which resulted in a multivariable OR of 12.52 (95% CI, 10.99–14.26). Using the ABW-based recommended dose, 38% of women and 25% of men had excess dosing, which led to a multivariable OR of 1.98 (95% CI, 1.81–2.15) (Table 2). Age group was not associated with the risk of excess dosing in crude comparison (Table 2); however, our sequential adjustment of covariates in our model revealed that this null association was confounded by the predominant presence of young women among HCQ users. As such, when our model was adjusted for female sex, older patients were more likely to be prescribed HCQ doses exceeding either recommendation in the adjusted models (multivariable OR 1.23 (95% CI 1.15–1.32) for IBW and multivariable OR 1.18 (95% CI 1.10–1.27) for ABW) (Table 2).

Proportions of initial HCQ excess dosing per the IBW-based recommendation increased with increasing BMI (28%, 35%, 36%, and 44% in underweight, normal, overweight, and obese categories, respectively) (Table 2). These resulted in a multivariable OR of 1.61 (95% CI 1.49–1.75) for obese individuals versus individuals with normal BMI (Table 2). In contrast, proportions of initial HCQ excess dosing per ABW-based recommendation decreased with increasing BMI (48%, 53%, 39%, and 10%, in underweight, normal, overweight, and obese categories, respectively). These resulted in a multivariable OR of 0.10 (95% CI 0.09–0.11) among obese individuals versus individuals with normal BMI. Patients with CKD had a lower risk of excess dosing by IBW (OR 0.88 (95% CI 0.73–1.00)) and by ABW (OR 0.87 (95% CI 0.76–0.99)).

Of 4276 (20%) subjects prescribed HCQ for 5 years or more, the dose at 5 years of use remained largely unchanged (median 400 mg/day). There was no difference in the proportion of excess dosing between the initial dose and dose at 5 years. Of these subjects, 40% and 37% had excess dosing by IBW for the initial dose and at 5 years (*p* = 0.40); 37% and 33% had excess dosing by ABW for the initial dose and at 5 years, respectively (*p* = 0.53).

## Discussion

In this UK general population-based cohort, we identified a modest decline in excess HCQ dosing in recent years. While this decline may reflect increased awareness over time of HCQ retinopathy risk, excess HCQ dosing remains substantial over the past decade according to prior IBW-based recommendations [2, 16, 17]. Applying the latest ABW-based dosing recommendations [19] also led to a considerable proportion of HCQ excess dosing, including more than 50% among patients with normal BMI. Overall, the excess dosing using either recommendation was more frequent among women, nearing 50% using IBW and 38% using ABW criteria and was less frequent among those with CKD. Moreover, proportions of excess HCQ dosing did not change with long-term use of HCQ (i.e., > 5 years). This further identifies a target population with the potential for the largest cumulative HCQ exposure and high risk of toxicity [18, 32–34].

To our knowledge, body weight and CKD are the patient factors most associated with risk of HCQ retinopathy [18]. The presence of concomitant CKD was associated with a slightly reduced risk of HCQ excess dosing. This may reflect HCQ dose adjustment in some patients with renal disease. Although there is no clear consensus on appropriate dose reduction in renal insufficiency [35], CKD was associated with more than doubling of the prevalence of HCQ retinopathy in the recent KPNC study [18]. Regardless, in our study found more than 30% of CKD patients’ prescriptions still exceeded recommended doses per the prior or latest guidelines, suggesting potentially considerable room for improvement in this population.

BMI categories had an opposite impact on the risk of excess dosing depending on which weight-based dosing guideline was applied [16, 20]. Following the latest ABW-based guideline, [19] more than 50% of individuals with normal BMI would have had HCQ excess dosing, compared to 10% of obese patients. In contrast, using the prior IBW-based guideline [2, 16, 27], 44% of obese patients and 37% of normal patients were exposed to excess dosing. This demonstrates the need for provider awareness that the newer guidelines reclassify excess dosing among a greater proportion of individuals with normal body weight.

We also found a higher risk of excess dosing among women, likely because HCQ 400 mg daily, which is the most commonly used dose, falls into the excess ABW dose range and IBW dose range among average-size women in the UK [36–38]. These findings suggest that prescribers in the UK may not have adjusted HCQ dosing based on IBW as recommended by the previous UK RCO guideline [16], particularly among women. It remains to be studied whether the new ophthalmology dosing guidelines will impact future prescribing patterns [20, 26]. Additionally, we found that older patients had approximately 20% higher risk of excess dosing than young patients (Table 2). As this is the first report on the potential impact of aging on excess dosing and its mechanism is not immediately clear, this finding awaits replication by future studies. It is also unknown whether women or older individuals have an increased risk of HCQ retinopathy, which could be correlated with these dosing findings.

A major strength of our study is the use of a large general population database to provide population-level data on HCQ prescribing patterns and thus our findings are likely to be generalizable. However, we were unable to directly address specialty prescriptions, although we found 68% of HCQ users were referred to rheumatologists and 9% to dermatologists around the time of first HCQ prescription, and results were similar in these users (data not shown). We focused on prescribed HCQ doses rather than assessing actual dispensed prescriptions or other measures of effective consumed dose of HCQ as our goal for this study was to assess prescribing patterns rather than patient adherence. Finally, our aim was to describe the level of excess HCQ dosing according to the previous and current guidelines; assessing the risk of retinal toxicity was beyond the scope of the current study. As the latest guideline change [20] has been largely based on the single large recent KPNC study [18], further data, particularly prospective evidence, are needed to accurately establish the risk of HCQ retinopathy and risk factors. We do not have sufficient patient-level clinical data to determine the clinical reasoning behind “excess” dosing. To that end, future studies should also address whether reducing HCQ dosing in many patients, following the existing ophthalmology guidelines, will retain the efficacy of this medication.

## Conclusions

In conclusion, this UK general population-based study found that according to previous and latest ophthalmology society guidelines, excess HCQ dosing is substantial, despite a modest decline over recent years. Female patients more often receive an excess HCQ dose according to these guidelines, and a similarly considerable proportion of long-term HCQ users also experience excess HCQ dosing. Over half of normal BMI individuals were excess-dosed per the latest guidelines; however, BMI had a noticeably opposite impact on the risk of excess dosing between the prior and latest weight-based guidelines. This implies the potential need to reduce dosing in many patients but also calls for further research to establish unifying, evidence-based safe-dosing strategies, balancing retinopathy risk and treatment efficacy.

## Additional file

Additional file 1: **Figure S1.** Overall hydroxychloroquine prescription dose trends. (DOCX 17 kb)
