# Supplementary material for: Hydroxychloroquine prescription trends and predictors for excess dosing per recent ophthalmology guidelines
Source: Arthritis Res Ther. 2018 Jul 5;20:133. doi: 10.1186/s13075-018-1634-8 (PMC6034317; doi:10.1186/s13075-018-1634-8)
Supplement: Supplementary file 1 — Figure S1. Overall hydroxychloroquine prescription dose trends. (DOCX 17 kb) [file 13075_2018_1634_MOESM1_ESM.docx]

**Figure S1:** **Overall Hydroxychloroquine Prescription Dose Trends**

Proportion of all hydroxychloroquine prescriptions (initial and ongoing) in each dosing category during each 6-month period between 2007 and 2016.
